# Protocol for the generation, viability assessment, and downstream processing of murine precision-cut skin slices

**DOI:** 10.1016/j.xpro.2026.104828

**Published:** 2026-09-10

**Authors:** Nicolas Perrard, Manel Jendoubi, Blanche Daunou, Farah Mana, Alexis Largy, Matthias Danes, Laurent Dubuquoy, Sylvain Dubucquoi, David Launay, Silvia Speca

**Affiliations:** 1 University of Lille, Inserm, CHU Lille, U1286 - INFINITE - Institute for Translational Research in Inflammation, 59000 Lille, France; 2 Department of Internal Medicine and Clinical Immunology, CHU Lille, 59000 Lille, France; 3 Institute of Immunology, CHU Lille, 59000 Lille, France

**Keywords:** Cell culture, Immunology, Organoids

## Abstract

Precision-cut tissue slices (PCTS) are an *ex vivo* model of viable organ sections widely used for therapeutic screening, toxicity assessment, and infection modeling. Here, we present a protocol for the generation, viability assessment, and downstream processing of murine Precision-cut Skin Slices (PCSS). We describe steps for preparing and agarose embedding skin samples, generating PCSS, maintaining them in culture, detecting lactate dehydrogenase (LDH), and quantifying ATP. We then detail procedures for RNA/protein extraction, the quantification of PCSS homogenates, and histological and TUNEL analysis of PCSS.

## Highlights

• Instructions for generation and maintenance of PCSS in culture
• Steps for viability assessment with LDH detection and ATP quantification
• Guidance on RNA/protein extraction, PCSS dissociation, and cellular count
• Procedures for histological and TUNEL analysis of PCSS

## Abstract (editor highlights)

Publisher’s note: Undertaking any experimental protocol requires adherence to local institutional guidelines for laboratory safety and ethics.

## Graphical abstract

**Figure:**
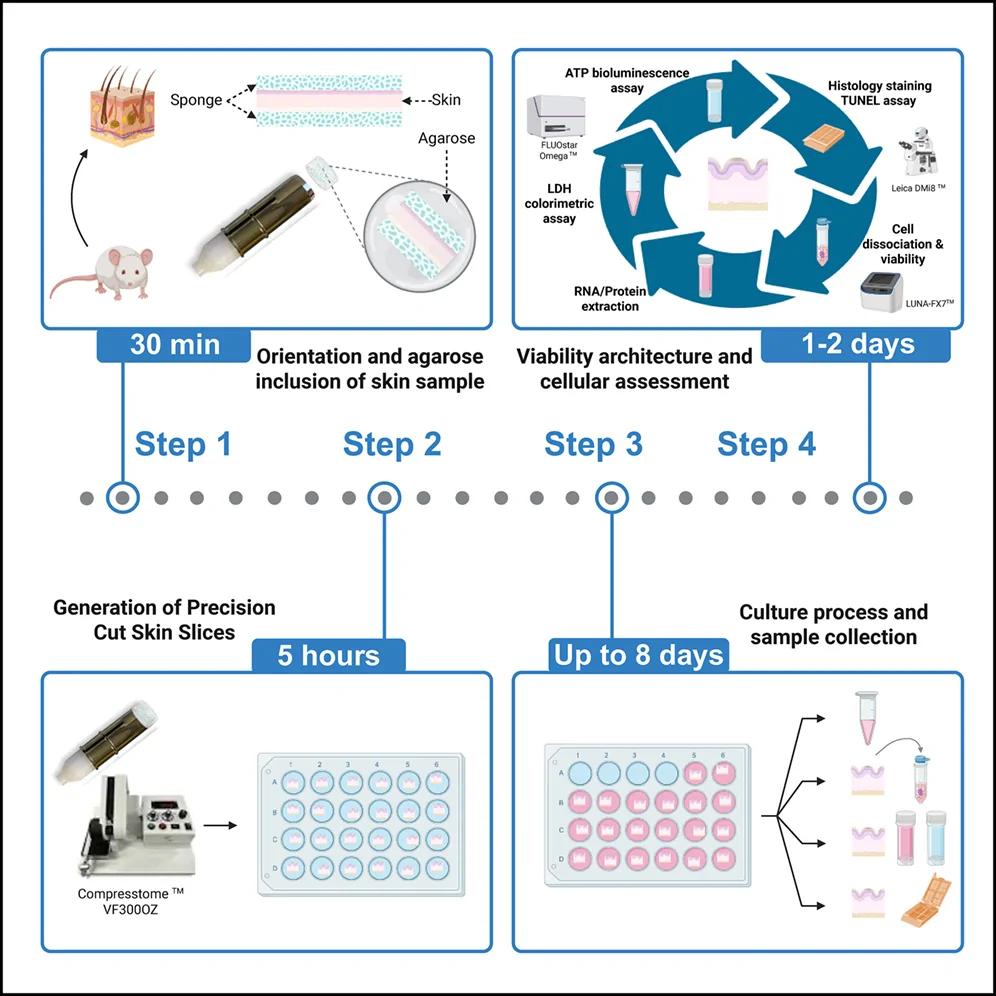

## Before you begin

### Innovation

Although Precision-Cut Tissue Slices (PCTS) have been described as a model for many years, the biomechanical properties of skin have thus far precluded the generation of Precision-Cut Skin Slices (PCSS). In this protocol, we present the technical innovations and optimizations of the standard PCTS protocol that enable th7e generation of murine skin slices. Improvements were also made to several standard protocols, such as the combined RNA/protein extraction protocol using TRIzol reagent, the enzymatic dissociation of the generated tissues, and the preparation of histological sections, for example.

### Media and solutions prepatation

**Timing: 1–2 h**

1. Prepare Dulbecco’s Modified Eagle Medium + supplements for PCSS culture as described in the “materials and equipment” section.

***Note:*** The 20% FBS medium is used only on the first day of culture, therefore a smaller volume can be prepared compared to the 10% FBS medium.

2. Add 500 μL of DMEM + supplements (20% FBS) to each well of two 24-well plates and place the plates in a humidified incubator at 37°C with 5% CO_2_ minimum 30 min before use.
3. Prepare 50 mL phosphate-buffered saline (PBS) pre-warmed at 37°C and 250 mL cold PBS kept at 4°C.
4. Melt 8% low-melting-point agarose in PBS until gentle boiling using a microwave oven and keep it at 42°C.

***Note:*** It may be necessary to adjust the temperature of the heating device depending on the mode of operation (water bath, dry bath, bead bath).

***Note:*** Agarose may be stored at 4°C for up to 2 weeks; however, repeated melting cycles may increase agarose concentration due to PBS evaporation. We recommend preparing a fresh agarose solution whenever possible.

5. Pre-warm the transfer pipette and the metal insert of the specimen holder at 42°C and maintain at this temperature until use (see Figure 1A).

**Figure 1:**
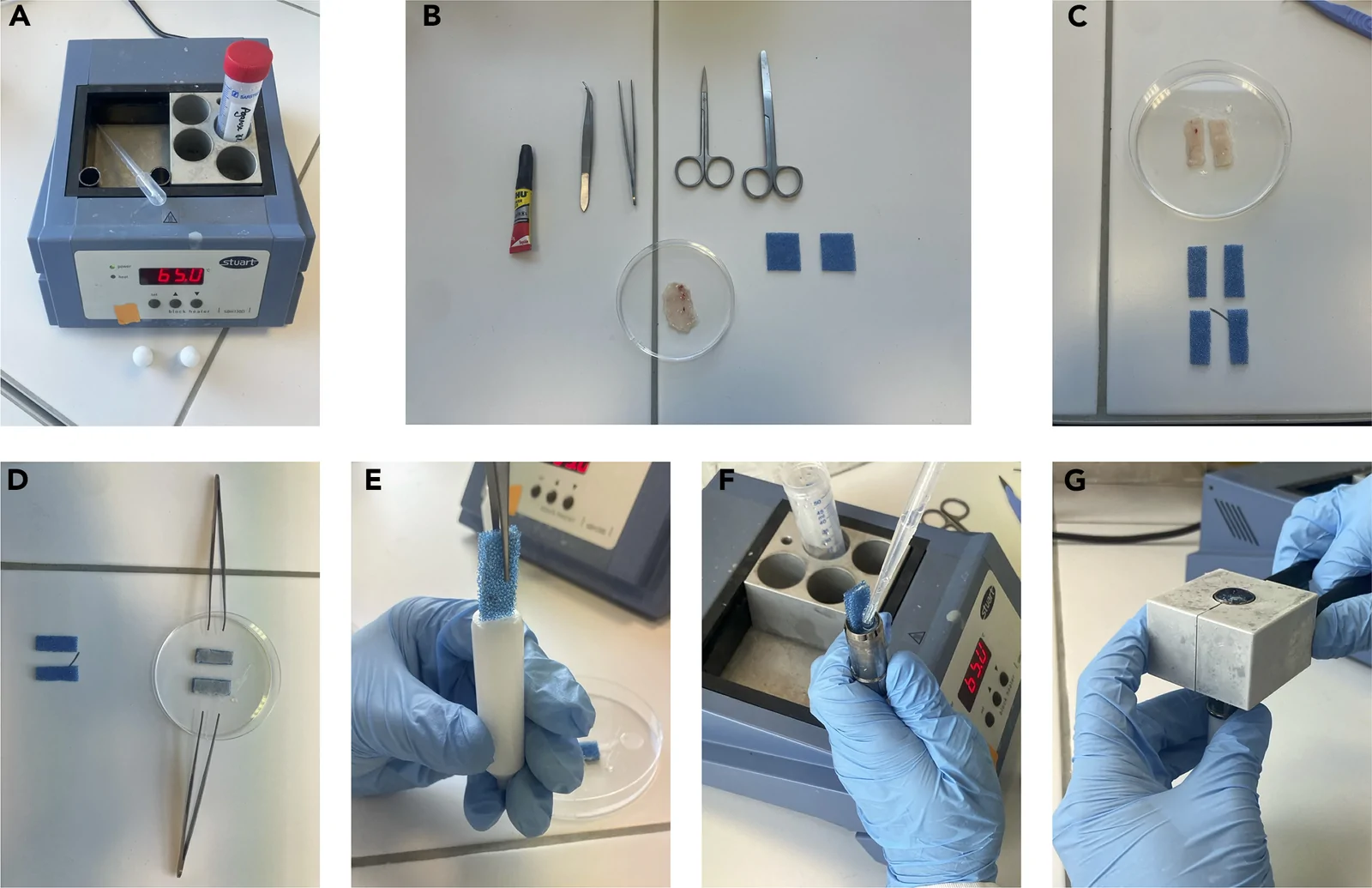
Photographic illustration of the specimen holder assembly described in the first major step (A) Maintenance of liquid agarose and equipment at 42°C (B) Uncalibrated skin sample with the various instruments used (C) Trimming of skin samples and foam pads for sandwich assembly (D) Placement of the skin sample with the hypodermal surface in contact with the foam pad (E) Fixation of the sandwich onto the specimen holder piston using liquid gluee (F) Embedding in liquid agarose using a pre-warmed transfer pipette, with uniform distribution around the metal insert (G) Solidification of the agarose and skin sample using a −20°C cooling block.
6. Store the cooling block at −20°C before use.
7. Prepare Tris-EDTA (TE) buffer as described in the materials and equipment section.
  a. Fill bead-beating tubes with four 2.8 mm zirconium beads and 500 μL of TE buffer.

***Optional:*** Perform this step only if ATP quantification is planned.

8. Prepare guanidine hydrochloride (GndCl) 7M solution by dissolving 6.687 g guanidine hydrochloride in 10 mL ddH_2_O.
  ***Note:*** If guanidine hydrochloride is difficult to dissolve, incubate in a water bath at 40 °C until fully dissolved, then filter through a 0.22 μm filter.
  a. Prepare resolubilization solution as described in the materials and equipment section.
  b. Fill bead-beating tubes with four 2.8 mm zirconium beads and 800 μL of TRIzol™.
    ***Optional:*** Perform this substep only if RNA/Protein isolation and quantification is planned.
9. Prepare collagenase/dispase solution as described in the materials and equipment section.

***Optional:*** Perform this step only if PCSS dissociation and cell count are planned.

10. Collect the mouse shaved skin sample according to the good practice recommendations of your institution and use it immediately in the protocol below.

***Note:*** If the sample cannot be used immediately, it may be stored in a PBS moistened gauze pad placed on a bed of ice for the shortest possible time. Do not immerse the skin sample in a solution, as the resulting skin edema will make PCSS preparation more difficult.

## Key resources table

**Table:** 

REAGENT or RESOURCE	SOURCE	IDENTIFIER
**Biological samples**		

Fetal Bovine Serum	Sigma-Aldrich	F7524-500ML

**Chemicals, peptides, and recombinant proteins**		

DMEM high glucose with GlutaMAX™ supplement	Gibco	10566016
HEPES	Gibco	15630080
Sodium pyruvate	Gibco	11360070
Non-Essential Amino Acids	Gibco	11140050
Penicillin-Streptomycin (10,000 U/mL)	Gibco	15140163
1x Dulbecco’s Phosphate Buffered Saline (DPBS)	Gibco	14190094
1x Hanks’ Balanced Salt Solution (HBSS)	Gibco	14025092
Collagenase, Type IV, powder	Gibco	17104019
Dispase, Type II, powder	Gibco	17105041
TRIzol™	Invitrogen	15596026
Chloroform	Sigma-Aldrich	1024441000
Agarose Low-Melting	Fisher BioReagents	BP1360-100
Trizma hydrochloride	Sigma-Aldrich	T2663
Ethylenediamine tetraacetic Acid 98% (EDTA)	Sigma-Aldrich	03620
Hydrochloric acid	Fisher Scientific	10316380
Sodium hydroxide	Fisher Scientific	10306240
Guanidine hydrochloride (GndCl)	Invitrogen	11528736
Urea	Sigma-Aldrich	U5128
Sodium dodecyl sulfate 20%	Euromedex	EU2-9194-5
Sodium pyrophosphate	Sigma-Aldrich	P8010
Sodium fluoride	Sigma-Aldrich	201154
Phenylmethanesulfonyl fluoride (PMSF)	Sigma-Aldrich	P7626
PhosSTOP™	Roche	4906837001
cOmplete mini™	Roche	11836153001
Triton X-100	Sigma-Aldrich	X100
Paraformaldehyde 4% pH 7	Microm Microtech	40877-36-MMF
Ethanol 100%	Microm Microtech	40820-36-MMF
2-propanol (Isopropanol)	Sigma-Aldrich	190764
Xylene	Microm Microtech	48974-36-MMF
ImmEdge Pen	Vector laboratories	H-4000
Eukitt classic	Orsatec GmbH	N/A
Fluoromount - Aqueous mounting medium	Sigma-Aldrich	F4680
Harris’ hematoxylin	Bio Optica	05-06004/L
Eosin Y 1%	Bio Optica	05-10007/L
Diawax paraffin 56-58°C	DiaPath	030980
Acridine Orange/Propidium Iodide Stain	Logos Biosystem	F23001

**Critical commercial assays**		

ATP Bioluminescence Assay Kit CLS II	Roche	11 699 695 001
Cytotoxicity Detection Kit (LDH)	Roche	11 644 793 001
Onestep TUNEL Apoptosis kit – [Red, 594]	Biotechne	NBP3-11959
NucleoSpin® RNA, Mini	Macherey-Nagel	740955
Pierce™ BCA Protein Assay Kit	Thermo Fisher	23225

**Experimental models: Organisms/strains**		

Mouse: BALB/c (8- to 15-week-old)	Janvier Labs	N/A

**Software and algorithms**		

GraphPad Prism 10.0	GraphPad Software	https://www.graphpad.com/
MARS Data Analysis Software	BMG Labtech	https://www.bmglabtech.com/en/microplate-reader-software/
LAS X Life Science Microscope Software Platform	Leica	https://www.leica-microsystems.com/products/microscope-software/

**Other**		

Transfer pipette	Sarstedt	86.1171.010
2.8mm Zirconium oxide beads	Precellys	KT03961-1-102.BK
Bead-beating tubes 2mL	Sarstedt	72.693.005
Dissecting case – Model B	Dutscher	005101
Petri dish 100x20 mm	Merck	P5606-400EA
Sterile disposable scalpel	Swann-Morton	0503
Cyanoacrylate liquid glue	UHU	N/A
Histology foam pads 30.2x25.4x2 mm	Simport	M476-1
TissueLoc Slotted Cassette	Epredia	B851954PK
Hi-stainless double edge razor blades	Feather	N/A
Block heater	Stuart	SBH130D
Water bath	Julabo	N/A
Cell incubator HERAcell 150	Thermo Scientific	16496629
Incubator PROLABO EB28	CAP-LAB	P21091244
Microwave oven	N/A	N/A
Compresstome VF-300OZ	Precisionary Instruments	N/A
Bead mill 24 homogenizer	Fisherbrand	15515799
Centrifuge for 1.5 mL tubes	N/A	N/A
Orbital shaker	Thermo Fisher	SK4000
FLUOstar Omega plate reader	BMG Labtech	N/A
LogosOne	Microm Microtech	N/A
HistoCore Multicut	Leica	N/A
HistoCore Arcadia C	Leica	N/A
HI1220 Flattening Table	Leica	N/A
Disposable blades	Microm Microtech	MM35+
TES Valida	MEDITE	N/A
Superfrost Plus Adhesion microscope slides	Epredia	J1800AMNZ
Superfrost microscope slides	Epredia	AG00008532E01MNZ20
Microscope slide holder	N/A	N/A
Cover slips 25x50 mm	Knittel glass	F/LCO25X50
Dmi 8 microscope	Leica	N/A
Multipette E3	Eppendorf	4987000010
Cell strainer 70 μm	Corning	CLS431751
LUNA™ Cell counting slides	Logos Biosystem	L12001
NanoDrop™ DS-11	DeNovix	N/A
LUNA-FX7™	Logos Biosystem	N/A

## Materials and equipment

### Mouse line

This protocol was established with shaved back skin from 8-15 weeks-old female BALB/c mice. Ensure that all mouse maintenance, handling and euthanasia must be carried out in accordance with all national and institutional animal welfare requirements and guidelines.

**Table:** Dulbecco’s modified Eagle’s medium (DMEM) + supplements (10 or 20% FBS)

Reagent	Final concentration	Stock concentration	Amount
HEPES	25 mM	1 M	12.5 mL
Sodium pyruvate	2 mM	100 mM	2 mL
Penicillin/Streptomycin	1% (v/v)	N/A	5 mL
FBS	10 or 20% (v/v)	N/A	50 or 100 mL
Non-Essential Amino Acids	1% (v/v)	N/A	5 mL
DMEM high glucose + GlutaMAX™	–	N/A	Up to 500 mL
**Total**			**500 mL**

Store at 4°C for up to 3 months

**Table:** TE buffer

Reagent	Final concentration	Amount
Trizma hydrochloride	100 mM	157.6 mg
Ethylenediamine tetraacetic Acid	4 mM	11.68 mg
Hydrochloric acid or Sodium hydroxide	pH 7.75	N/A
ddH_2_O	–	100 mL

Store at 4°C for up to 3 months

***Optional:*** Prepare a 10x concentration and store it for up to 1 year at 4°C.

**Table:** Resolubilization solution

Reagent	Final concentration	Stock concentration	Amount
Urea	8 M	N/A	961 mg
Trizma hydrochloride	40 mM	1 M	80 μL
Sodium dodecyl sulfate	1% (v/v)	20% (v/v)	100 μL
Sodium pyrophosphate	2 mM	0.2 mM	20 μL
Sodium fluoride	100 mM	0.5 M	400 μL
Phenylmethanesulfonyl fluoride (PMSF)	1 mM	1 M	20 μL
Ethylenediamine tetraacetic Acid (EDTA)	5 mM pH 8	0.5 M	20 μL
PhosSTOP™ (1 pellet in 1 mL ddH_2_0)	1x	10x	200 μL
cOmplete mini™ (1 pellet in 1.5 mL ddH_2_0)	1x	7x	285 μL
ddH_2_O	N/A		Up to 2 mL
**Total**			**2 mL**

Prepare this solution freshly before each use; do not store (urea is unstable).

**Table:** Collagenase/Dispase solution (1x)

Reagent	Final concentration	Amount
Collagenase IV	1 mg/mL	4 mg
Dispase II	0.4 mg/mL	1.6 mg
HBSS	N/A	4 mL

Store at 4°C for up to 1 week.

***Optional:*** Prepare a 10x concentration and store it for up to 1 year at −20°C.

**Table:** Vibratome settings

Parameters	Settings
Vibratome model	Compresstome™ VF-300OZ (Precisionnary Intruments®)
Agarose LMP concentration	8% w/v
Vitesse	4 to 6
Oscillation	4
Agarose solidification time	15 min at 4°C
PCSS thickness	500 μm
PCSS length	1 cm
PCSS generation/Agarose dissociation temperature	4°C/37°C
Skin sample orientation	Longitudinal

LMP: low-melting point

**Table:** Fisherbrand™ homogenizer Bead Mill 24 settings

Parameters	Settings
Speed	4 m/s
Cycle	4
Cycle duration	40 s
Pause time	60 s

## Step-by-step method details

### Preparation and agarose embedding of the skin sample

**Timing: 30 min**

The biomechanical properties of skin make sectioning challenging. Thus, next steps are critical for successful PCSS preparation, as well as for maintaining PCSS viability during extended culture (see troubleshooting 1). Substeps are illustrated in Figure 1. It is recommended that these steps are performed jointly by two operators to facilitate handling.

1. Place the skin sample flat in a Petri dish and use a scalpel and scissors to carefully remove superfluous anatomical elements (fascia, fat, hair, etc.).

***Optional:*** Shaving and removal of superfluous anatomical elements may be performed on the animal during skin sample collection.

2. Use a scalpel to cut the skin sample into a rectangle of no more than 12 × 22 mm (see Figure 1C).

***Note:*** Exceeding these dimensions may cause difficulty in correctly embedding the assembly in agarose (width > 12 mm) or prevent mounting the specimen holder on the instrument (length > 20 mm). For this protocol, we recommend preparing a 10 x 20 mm skin sample.

***Optional:*** If performing the histological analyses described below, preserve a control mouse skin sample between two foam pads in a histology cassette in a 4% buffered paraformaldehyde (PFA) solution at 20°C.

***Note:*** PCSS in 4% buffered PFA may be stored at 4°C for up to 24 h before paraffin embedding. If storage lasts longer than 24 h, replace PFA with PBS to avoid over-fixation.

3. Mount the skin sample between two histology foam pads (see Figure 1D).
  a. Cut two histology foam pads to the dimensions of the skin sample, not exceeding 12 × 22 mm.
  b. Carefully place the hypodermal face of the skin sample on one foam pad and spread the edges of the sample as flat as possible so that it lies as straight as possible on the foam.
  c. Place the second foam pad on the epidermal surface of the skin sample and press gently to form a compact sandwich.

**CRITICAL:** This skin mounting procedure is essential for PCSS generation using the Compresstome™

4. Glue the sandwich to the base of the specimen holder piston (see Figure 1E)
  a. Place a drop of cyanoacrylate liquid glue on the Petri dish.
  b. Grip the sandwich with flat-tipped forceps and briefly dip the end into the glue drop.
  c. Place the glued end of the sandwich onto the specimen holder piston and wait a few seconds for the glue to set.
5. Embed the sandwich in 8% liquid agarose solution (see Figure 1F)
  a. Insert the specimen holder piston into the metal insert pre-warmed at 42°C.
  b. Using a transfer pipette, rapidly collect the 8% liquid agarose maintained at 42°C and deposit it evenly between the sandwich and the metal insert.
  c. Progressively raise the metal insert until the sandwich is completely embedded.

**CRITICAL:** Before proceeding to the next step, ensure that the agarose is evenly distributed and that no air bubbles are present between the sandwich and the metal insert, particularly along the lateral edges. This is critical for sectioning the skin sample along its entire length (see troubleshooting 1).

6. Insert the specimen holder into the −20°C cooling block for 1 min 30 s to superficially solidify the agarose (see Figure 1G)
7. Store the specimen holder containing the skin sample at 4°C for 15 min, then proceed to the next step.

### PCSS generation and maintenance in culture

**Timing: 6 h**

**Figure 2:**
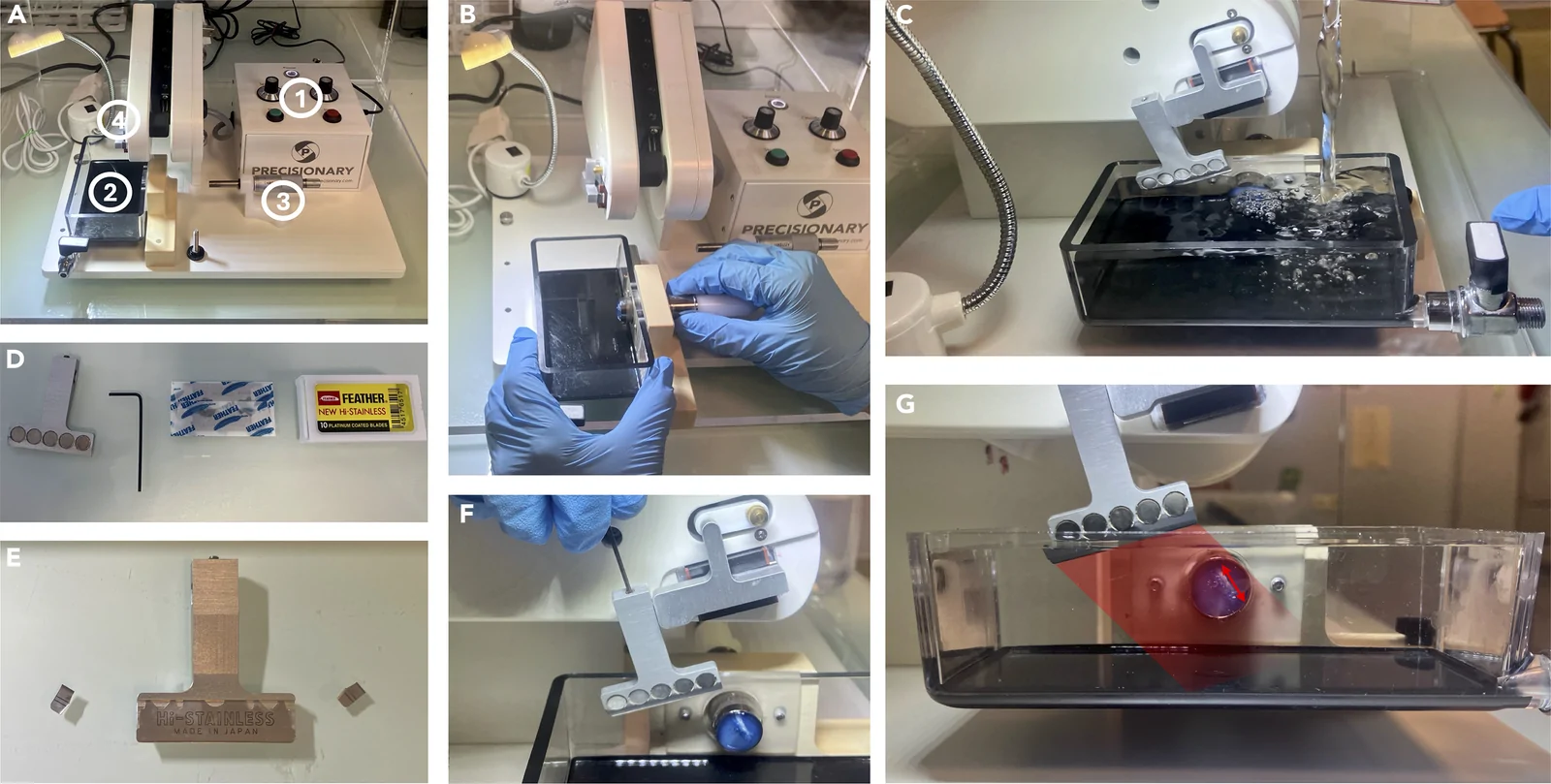
Photographic illustration of PCSS generation described in the second major step (A) General view of Compresstome VF-300OZ™, with ① Control panel, ② Collection tray, ③ Blade holder’s vibrating inserter and ④ Micrometer screw (B) Insertion of the specimen holder into the collection tray (C) Filling the collection vessel with 4°C PBS until complete immersion of the sample holder (D) From left to right: blade holder, tightening key, and Feather® razor blades (E) Use of a Feather® half-blade, then fitting it onto the blade holder after removing the ends (F) Securing the blade holder on the vibrating inserter using the tightening wrench. (G) Front view of the sample holder, showing the sandwich (red double arrow) oriented perpendicular to the path of the vibrating blade (transparent red rectangle).

This step involves preparing the PCSS using the Compresstome™ and initiating culture. Substeps are illustrated in Figure 2. It is important to work as possible under sterile conditions to minimize contamination (for further information, see troubleshooting 2). After generation, culture media are replaced every 24 h.

8. Prepare the Compresstome™ for PCSS sectioning.
  a. Switch on the instrument, remove the collection tray, and fully unscrew the micrometer screw (see Figure 2A).
  b. Insert the cold specimen holder into the designated location in the transfer trough, then secure the trough to the instrument by tightening the retaining screw fully (see Figure 2B).
  c. Fill the transfer trough with cold PBS (4°C) until the specimen holder is completely submerged (see Figure 2C).
  d. Mount a half ultra-sharp razor blade on the magnetic blade holder, insert the blade holder into its designated location, and tighten the retaining screw fully (see Figures 2D–2F).
  e. Rotate the specimen holder piston to orient the sandwich perpendicular to the blade cutting axis (see Figure 2G).
  f. Tighten the micrometer screw until it contacts the piston.

***Note:*** If the blade holder is not magnetic, secure the razor blade with liquid glue.

**CRITICAL:** Ensure the specimen holder remains fully submerged in cold PBS to preserve tissue integrity and facilitate the sectioning process.

9. Set the Compresstome™ parameters as described in the materials and equipment section.

***Note:*** The cutting speed may initially be increased before reaching the skin sample.

10. Turn the micrometer screw one full turn (500 μm), then press “Start” (see Methods Video S1: Generation of PCSS using Compresstome™ VF-300OZ).
  a. If the disc in the collection tray contains only agarose or foam, discard it.
  b. If the disc contains tissue between the foam pads, gently transfer it to a Petri dish containing PBS at 37°C.
  c. Separate the two foam pads to isolate the PCSS using two flat-tipped forceps, then transfer it to a well of the 24-well culture plate previously filled with 500 μL of DMEM + supplements with 20% FBS (see Figure S1, Illustration of different steps of removing PCSS from agarose embedding and foam pad sandwich).
  d. Repeat substep 10 until the skin sample is exhausted, as indicated by the presence of glue in the foam pads and on the skin sample.

***Note:*** It is advisable to discard the first PCSS prepared, as well as the last sections that show the presence of glue.

**CRITICAL:** Orientation is critical for skin sample sectioning. If you experience difficulties cutting the skin sample, see troubleshooting 1.

11. Place the 24-well culture plate containing the sections in a humidified incubator at 37°C with 5% CO_2_ for 5 h.

**CRITICAL:** This step removes toxic byproducts generated by cell death induced by the sectioning process.

12. Aspirate the medium from each well carefully, then rinse each well with 500 μL of PBS pre-warmed at 37°C and replace it with 500 μL of DMEM + supplements with 10% FBS pre-warmed at 37°C.

***Note:*** PCSS may occasionally adhere to the plastic tip of the pipette or to the well wall when medium is removed. If this occurs, see troubleshooting 3.

13. Return the 24-well culture plate containing sections to the incubator at 37°C with 5% CO_2_.

**Pause point:** PCSS can be maintained in culture for 24 h without any significant mortality. Repeat sub-steps 12 and 13 every 24 h for up to 8 days.

Methods Video S1. Generation of PCSS using Compresstome

### LDH detection in culture media

**Timing: 1 h**

Lactate dehydrogenase (LDH) is a ubiquitous intracellular enzyme essential for sugar metabolism. Its increase in culture media indicates cellular stress and mortality. The LDH assay is a simple and non-destructive method to monitor PCSS damage and cytotoxicity over time, directly in the culture medium and without scarifying the samples. This step allows indirect assessment of cell death within the PCSS.

14. Collect media designated for the detection of lactate dehydrogenase (LDH) enzyme.
  a. Prepare a solution of 20% Triton X-100 by adding 50μL of Triton in 200 μL of culture medium which will be used as a positive control for PCSS mortality.
  b. Add 50 μL of the 20% solution to designated positive control wells for a final concentration of 2% Triton X-100 and incubate the PCSS for 10 min.
    ***Note:*** The addition of 2% Triton X-100 to the culture medium permeabilizes the cell membranes of the sections and maximizes LDH release into the medium.
  c. Collect the culture media from each well into individual Eppendorf tubes and discard the two PCSS incubated in Triton X-100.
  d. Centrifuge the culture media collected before at 500 × *g*, 4°C for 5 min.
  e. On ice, carefully collect the culture media into new Eppendorf tubes, avoiding the lipid-containing supernatant and the cell debris pellet.
    ***Note:*** This sub-step is particularly important during the first days of culture due to the mortality induced by the sectioning process. The presence of lipids and cell debris in the culture media may interfere with LDH detection.
    **Pause point:** Culture media may be stored at −20°C for up to 1 week or at −80°C for up to 1 year before performing assays.
15. Reconstitute the lyophilized dye in 1 mL of ddH_2_O for 10 min. Thaw the catalyst on ice before use.
16. Add 100 μL of culture medium previously recovered as in step 15 in duplicate to the wells of a 96-well plate.

***Note:*** Replace media by DMEM + supplements (10% FBS) for negative control and Triton X-100-treated culture medium for positive control

***Note:*** Culture media can generally be used undiluted or diluted depending on sample size or culture volume to ensure that LDH levels fall within the linear range of the assay. Include positive controls in duplicate and diluted if necessary.

17. Prepare the reaction mixture: combine 250 μL of dye solution with 11.25 mL of catalyst per 100 assays.

***Note:*** The Reaction mixture must be freshly prepared for each assay.

18. Add 100 μL of freshly prepared reaction mixture to each well and incubate in the dark for 10 min.
19. Measure the absorbance of each sample at 490 nm using a FLUOstar Omega™ plate reader or equivalent reader.

### ATP quantification in PCSS homogenates in TE buffer

**Timing: 1 h**

Adenosine triphosphate (ATP) is a nucleotide that provides the energy required for metabolic chemical reactions, cell division, and the active transport of chemical species across biological membranes. It serves as an indicator of metabolism and cell viability. The ATP bioluminescence is a method to measure cell metabolism and viability directly on the PCSS. In this protocol, we performed this method to validate the LDH measurements in the culture medium.

20. Collect the PCSS designated for ATP quantification.
  a. Aspirate the medium from each well carefully, then rinse each well with 500 μL of PBS pre-warmed at 37°C.
  b. Collect one PCSS in 500 μL of TE buffer directly into bead-beating tubes containing four 2.8 mm zirconium beads, prepared in advance.
  c. Homogenize the PCSS collected in bead-beating tubes using a Fisherbrand® Bead Mill 24 homogenizer according to the parameters described in the materials and equipment section.
  d. Incubate for 5 min at 20°C in the homogenizer, then repeat substep 20.c a second time.
  e. Centrifuge the bead-beating tubes at 1,000 × *g* at 4°C for 1 min.
  f. On ice, carefully collect the TE-homogenate into an Eppendorf tube, avoiding the lipid-containing supernatant and the pellet of cell and tissue debris.

**Pause point:** PCSS homogenates in TE buffer may be stored at −20°C for up to 1 week or at −80°C for up to 1 year before performing assays.

21. Reconstitute the Luciferase Reagent in 10 mL of ddH_2_O and incubate for 5 min at 4°C without stirring or shaking.
22. Add the appropriate volume of ddH_2_O to the ATP Standard vial to achieve a final concentration of 10 mg/mL (16.5 mM).

***Note:*** We recommend aliquoting the ATP Standard to prevent degradation from repeated freeze-thaw cycles and to ensure reproducibility across experiments.

**CRITICAL:** All steps involving ATP measurements must be performed on ice to prevent ATP degradation.

23. Prepare ATP standard dilutions from 10^-4^ to 10^-11^ M by adding 10 μL of ATP standard to 1,640 μL of ddH_2_O (10^-4^ M), then perform serial 1:10 dilutions by transferring 100 μL of the previous solution into 900 μL of ddH_2_O down to 10^-11^ M.
24. Add 50 μL of each TE-homogenate sample in duplicate to the wells of a 96-well plate.

***Note:*** Replace samples by ATP Standard dilutions in duplicates for calibration, and TE buffer for negative control.

***Note:*** TE-homogenate sample can generally be used undiluted

25. Add 50 μL of Luciferase Reagent to each well and begin measuring the luminescence of each sample immediately using the FLUOstar Omega™ plate reader or equivalent reader.

**CRITICAL:** Ideally, use the automatic injection function of the plate reader to avoid measurement variability caused by the rapid loss of luciferase luminescent activity.

**Alternative:** Use an automatic reservoir dispenser to add Luciferase Reagent with minimal delay.

### RNA/protein extraction and quantification in PCSS homogenates in TRIzol

**Timing: 7 h**

This step describes an adaptation of the mixed extraction protocol using TRIzol™, optimized for PCSS. Individual PCSS are used for RNA extraction and PCSS are pooled in pairs for protein extraction. RNA extracted using this method is compatible with numerous downstream applications, whereas the use of extracted proteins is restricted to a limited number of applications (e.g., Western blotting or mass spectrometry). The nature of the proteins obtained through this extraction procedure is not compatible with techniques requiring native, non-denatured proteins (e.g., ELISA).

TRIzol™ is a hazardous reagent that must be handled with appropriate collective and individual protective equipment in accordance with institutional safety guidelines.

26. Collect the PCSS designated for RNA/protein extraction.
  a. Aspirate the medium from each well carefully, then rinse each well with 500 μL of PBS pre-warmed at 37°C.
  b. Collect one PCSS in 750 μL of TRIzol™ directly into bead-beating tubes containing four 2.8 mm zirconium beads, prepared in advance.
  c. Homogenize the PCSS collected in bead-beating tubes using a Fisherbrand® Bead Mill 24 homogenizer according to the parameters described in the materials and equipment section.
  d. Incubate for 5 min at 20°C on the homogenizer, then repeat substep 26.c a second time.

**Pause point:** PCSS homogenates in TRIzol™ may be stored at −20°C for up to 1 year before performing assays.

27. Separate RNA from proteins within the TRIzol™–homogenate.
  a. Incubate for 30 min at −70°C, then 5 min at 60°C, then 5 min on ice.
  b. Transfer the TRIzol™–homogenate to a 2 mL Eppendorf tube.
  c. Add 150 μL of chloroform.
    ***Note:*** Chloroform may be substituted with 1-bromo-3-chloropropane, which is less toxic.
  d. Vortex for 10 s, then incubate for 3 min at 20°C.
  e. Centrifuge at 12,000 × *g* at 4°C for 15 min.
  f. Collect the upper aqueous phase containing RNA into a 2 mL Eppendorf tube and proceed to step 28 for RNA extraction.
  g. Discard the white interphase and collect the lower organic phase containing proteins into a 2 mL Eppendorf tube.
    **Pause point:** The organic phase may be stored at 4°C for 24h or on ice until processing through sub-step 29.
28. Wash and isolate RNA.
  ***Note:*** For this sub-step, replace the collection tube after each centrifugation.
  a. Add 350 μL of 70% ethanol to the aqueous phase obtained in step 27.f and mix by pipetting.
  b. Transfer the precedent mix onto a Macherey-Nagel column (blue), then centrifuge at 11,000 × *g* at 4°C for 1 min.
  c. Add 350 μL of Membrane Desalting Buffer (MDB), then centrifuge at 11,000 × *g* at 4°C for 1 min.
  d. Reconstitute the DNase by adding 550 μL of RNase-free H_2_O to the lyophilized DNase, then prepare a 1:10 DNase solution in Reaction Buffer for rDNase.
  e. Add 95 μL of the 1:10 DNase solution to the Macherey-Nagel column and incubate for 15 min at 20°C.
  f. Add 200 μL of RAW2 buffer, then centrifuge at 11,000 × *g* at 4°C for 30 s.
  g. Add 600 μL of RA3 buffer, then centrifuge at 11,000 × *g* at 4°C for 30 s.
  h. Add 250 μL of RA3 buffer, then centrifuge at 11,000 × *g* at 4°C for 2 min.
  i. Transfer the Macherey-Nagel column to a 1.5 mL collection tube, add 40 μL of RNase-free H_2_O, then centrifuge at 11,000 × *g* at 4°C for 1 min.
29. Quantify RNA using a NanoDrop™ DeNovix DS-11 spectrophotometer or equivalent.
30. Wash and isolate proteins.
  a. Add 300 μL of 100% ethanol to the organic phase obtained in step 27g to precipitate DNA, and mix by inversion.
  b. Incubate for 3 min on ice, then centrifuge at 2,000 × *g* at 4°C for 5 min.
  c. Carefully transfer the supernatant to a new 2 mL Eppendorf tube, avoiding the DNA pellet.
  d. Add 1.5 mL of isopropanol and mix by inversion.
  e. Incubate for 60 min at −80°C, then centrifuge at 12,000 × *g* at 4°C for 10 min.
  f. Aspirate the supernatant with a pipette, then allow the protein pellet to air-dry for 5 min inverted on clean absorbent paper.
  g. Resuspend the pellet in 100 μL of 7 M GndCl solution and incubate for 10 min.
  h. Pool proteins from pairs of PCSS to obtain 200 μL of 7 M GndCl solution.
  i. Add 1.8 mL of 100% ethanol and vortex for 10 s.
  j. Incubate for 60 min at −80°C, then centrifuge at 12,000 × *g* at 4°C for 5 min.
  k. Aspirate the supernatant.
    **CRITICAL:** If the pellet still appears red, resuspend it in 200 μL of GndCl and repeat steps 30.i, 30.j, and 30.k.
  l. Resuspend the pellet in 2 mL of 100% ethanol.
  m. Vortex for 10 s, agitate for 5 min, vortex again for 10 s, then centrifuge at 12,000 × *g* at 4°C for 10 min.
  n. Aspirate the supernatant with a pipette, then allow the protein pellet to air-dry for 5 min inverted on clean absorbent paper.
  o. Add 100 μL of Tris/Urea/SDS resolubilization solution.
  p. Vortex for 10 s, agitate for 5 min on an orbital shaker, vortex again for 10 s, then collect the resolubilized protein solution.
    ***Note:*** Protein resolubilization may be incomplete or difficult with the persistence of a solid precipitate, then see troubleshooting 4.
    **Pause point:** Protein samples may be stored at −20°C for up to 1 week or at −80°C for long-term storage before performing assays.
31. Protein quantification using the Pierce™ BCA Protein Assay Kit according to the standard procedure.

***Note:*** Protein samples can generally be used undiluted or diluted to ensure concentrations fall within the linear range of the assay. We recommend including duplicates diluted 1:5.

32. Measure the absorbance of each sample at 562 nm using a FLUOstar Omega™ plate reader or equivalent.

### PCSS dissociation and cellular count

**Timing: 30 min**

This step allows PCSS cell suspension, as well as direct count and assessment of cell death within the PCSS using propidium iodide.

33. Collect the PCSS designated for tissue dissociation and cellular count.
  a. Carefully aspirate the medium from each well, then rinse each well with 500 μL of PBS pre-warmed to 37°C.
  b. Transfer the PCSS to a Petri dish and cut them into 2 mm pieces using a scalpel.
  c. Dissociate the PCSS in 4 mL of 1x collagenase/dispase solution at 37°C in a water bath overnight (approximatively 16 h).

***Note:*** To obtain sufficient cells for potential additional analyses, we recommend pooling PCSS in pairs in the collagenase/dispase solution.

34. Retrieve the dissociated PCSS from the water bath and add 1 mL of FBS.
35. Homogenize by pipetting, then filter the suspension through a 70 μm cell strainer pre-wetted with culture medium.

***Note:*** It is possible to observe the persistence of dermal collagen aggregates in the solution even after homogenization.

36. Centrifuge at 500 × *g* at 20°C for 5 min and discard the supernatant.
37. Resuspend the cell pellet in 1 mL of DMEM + supplements with 10% FBS.
38. Transfer 18 μL of the cell suspension to a 0.5 mL Eppendorf tube and add 2 μL of Acridine Orange/Propidium Iodide.
39. Load 10μL of the precedent mix on a cell counting slide and count total cells/assess viability using a LUNA-FX7™ fluorescence cell counter or equivalent device.

***Note:*** If the cellular count is especially low, or with a widely impaired viability, see troubleshooting 5.

### Standard histological analysis of PCSS

**Timing: 3 days**

Standard histological stainings are used to assess tissue integrity and confirm the preservation of the overall PCSS architecture during the culture process. It involves numerous hazardous chemicals (xylene, isopropanol, paraformaldehyde, acidic dyes) that must be handled with appropriate collective and individual protective equipment in accordance with specific institutional safety guidelines.

40. Collect the PCSS designated for standard histological or TUNEL analysis.
  a. Collect one PCSS and place it between two foam pads.
    **CRITICAL:** It is essential to position the PCSS as flat as possible between the foam pads in the cassette, to orient the epidermis above the dermis and to avoid twisting the PCSS to facilitate subsequent paraffin embedding (see the corresponding substep below and associated troubleshooting 6).
  b. Place the two foam pads containing the PCSS in histology cassettes and store in 4% buffered PFA solution.
    ***Note:*** PFA must be handled under a chemical fume hood; it is important to verify the compatibility of this product with the biosafety cabinet used for PCSS culture.
  c. Proceed to standard histological or TUNEL analysis step.
    **Pause point:** PCSS in 4% buffered PFA may be stored at 4°C for up to 24 h before paraffin embedding. If storage lasts longer than 24 h, replace PFA with PBS to avoid over-fixation.
41. Perform dehydration and paraffin impregnation of the PCSS and control mouse skin using standard tissue processing (dehydrate progressively in graded ethanol, clear with xylene and infiltrate tissue with molten paraffin wax)

***Note:*** Standard manual procedures or a Logos One™ type automated dehydration system can be used.

42. Retrieve the dehydrated and paraffin-impregnated cassettes and proceed to embedding.
  a. Open the cassettes, kept warm at 56°C, and discard the foam pads and cassette lid, retaining the PCSS and the cassette base.
  b. Fill the bottom of a metal mold with hot paraffin.
  c. Place the PCSS at the bottom of the mold, then transfer the mold to the −14°C cold plate to allow the paraffin to solidify.
  d. Fill the remaining volume of the mold with hot paraffin and place the cassette base on top.
  e. Place the metal mold on a cold plate and allow the paraffin to solidify for at least 30 min.
  f. Unmold the solidified paraffin block containing the PCSS and trim any excess paraffin from the external sides of the cassette.

**CRITICAL:** The orientation of the PCSS in substep 42.c is critical for subsequent steps and depends on the correct execution of substep 40.a. The PCSS must be positioned flat at the bottom of the mold with a clear visual distinction between the epidermis and the dermis (see troubleshooting 6)

**Pause point:** Paraffin blocks may be stored at room temperature (approximatively 20°C) indefinitely before use.

43. Prepare Superfrost™ microscope slides with 4 μm PCSS and mice skin histological sections using a microtome.
  a. Coat Superfrost™ microscope slides with a thin layer of ddH_2_O.
  b. Starting from blocks cooled to −14°C, carefully trim with 20 μm sections until the full PCSS surface is visible.
  c. Cut 4 μm sections and place them onto the water-coated slides.
  d. Transfer the slides to a 37°C hot plate for a few minutes, then drain the water.

***Optional:*** Prepare sections on Superfrost™ Plus Adhesion microscope slides for TUNEL analysis if needed

44. Place the dry Superfrost™ microscope slides in a vertical position in an incubator at 37°C for 24 h.

***Optional:*** Incubate for 48 h for TUNEL analysis (see corresponding step above).

45. Rehydrate the PCSS sections and perform Hematoxylin-eosin staining according to the standard procedure.
46. Dehydrate and mount slides with Eukitt® Quick-hardening mounting medium.

**Pause point:** Dried stained slides may be stored indefinitely before analysis.

47. Proceed to microscopic analysis of the slides using a Leica® Dmi 8™ microscope or equivalent microscope.

### TUNEL analysis of PCSS

**Timing: 3 h**

The TUNEL assay is used to detect specific and/or localized cell death within the PCSS by binding fluorescent component to double-strand DNA breaks generated during apoptosis or necrosis. We employed this technique to investigate the presence of cell-subtype-specific mortality inherent to the slicing or culture process.

It must be performed on Superfrost™ Plus Adhesion microscope slides prepared from sub-step 43 described above.

48. Rehydrate the PCSS sections as described in sub-step 45
49. Permeabilize the PCSS sections.
  a. Dry the slide with absorbent paper and encircle the PCSS on the slides using a hydrophobic pen before staining to confine reagents.
    ***Note:*** Ensure the ink is completely dry before adding aqueous solutions.
  b. Prepare a 1× Proteinase K solution by diluting the 100× Proteinase K stock 1:100 in PBS, then vortex.
  c. Add 50 μL of 1× Proteinase K solution to each PCSS.
  d. Incubate for 20 min at 37°C for optimal permeabilization.
50. Prepare positive and negative controls from control mouse skin sections.
  a. Prepare 1× DNase I buffer by diluting the 10× DNase I buffer 1:10 in ddH_2_O, then vortex.
  b. Prepare DNase I at 200 U/mL in 1× DNase I buffer.
    ***Note:*** Do not vortex DNase I, as this may destroy the enzyme.
  c. Prepare the positive control:
    i. Incubate the section with 50 μL of 1× DNase I buffer for 5 min at 20 °C.
    ii. Dry the slide with absorbent paper, then add 50 μL of DNase I solution at 200 U/mL.
    iii. Incubate for 30 min at 37°C.
    iv. Wash in 3 PBS baths for 5 min each.
  d. Prepare the negative control:
    i. Incubate the section with 50 μL of 1× DNase I buffer for 5 min at 20°C.
    ii. Dry the slide with absorbent paper, then add 50 μL of 1× DNase I buffer.
    iii. Incubate for 30 min at 37°C.
    iv. Wash in 3 PBS baths for 5 min each.
      ***Note:*** PCSS sections are kept in PBS at 20°C during controls preparation.
51. Perform TUNEL labeling.
  a. Prepare the TUNEL labeling solution by combining, per section and positive control: 35 μL of TdT Equilibration Buffer, 10 μL of Labeling Solution, and 5 μL of TdT enzyme.
  b. Prepare the TUNEL labeling solution for the negative control by combining, per negative control: 40 μL of TdT Equilibration Buffer and 10 μL of Labeling Solution (without TdT enzyme).
  c. Incubate each section and control with 50 μL of TdT Equilibration Buffer for 30 min at 37°C.
  d. Dry the slide with absorbent paper, then add 50 μL of TUNEL Labeling Solution. The negative control is incubated with TUNEL Labeling Solution without TdT enzyme.
  e. Incubate for 60 min at 37°C in a humidified chamber in the dark.
  f. Wash in 3 PBS baths for 5 min each in a light-tight container.
52. Nuclear staining with DAPI.
  a. Dry the slide with absorbent paper and incubate with DAPI solution for 5 min at 20°C in the dark.
  b. Wash in 3 PBS baths for 5 min each in a light-tight container.
53. Mount slides with Anti-Fluorescence Quenching Agent.
  a. Apply a drop of Anti-Fluorescence Quenching Agent to each PCSS, then place a glass coverslip on the slide.
  b. Remove air bubbles between the slide and coverslip.

**Pause point:** Labeled slides may be stored in the dark at 4°C for up to 1 week before microscopic analysis.

54. Proceed to microscopic analysis of the slides using a Leica® Dmi 8™ microscope or equivalent microscope equipped with two lasers and appropriate fluorescence filters (DAPI: ex. 350 nm/em. 470 nm; TRITC Red: ex. 590 nm/em. 617 nm).

## Expected outcomes

The main challenge facing skin models currently developed for the study of cutaneous pathologies lies in striking a balance between model complexity (which allows closer approximation of native skin complexity and greater physiological relevance) and model cost, standardization, and interlaboratory reproducibility.^1^ At present, simpler models (reconstructed epidermis, classical organotypic cultures) remain the preferred choice owing to their reproducibility, rapid turnaround, and controlled cost, at the expense of the biological fidelity offered by more complex models (iPSC-derived cells, bioengineering approaches such as 3D bioprinting or microfluidic systems).^2^

PCSS generated according to the standardized protocol described above yield a simple, viable, versatile architectural and organotypic model compatible with a broad range of downstream applications.^3^

**Figure 3:**
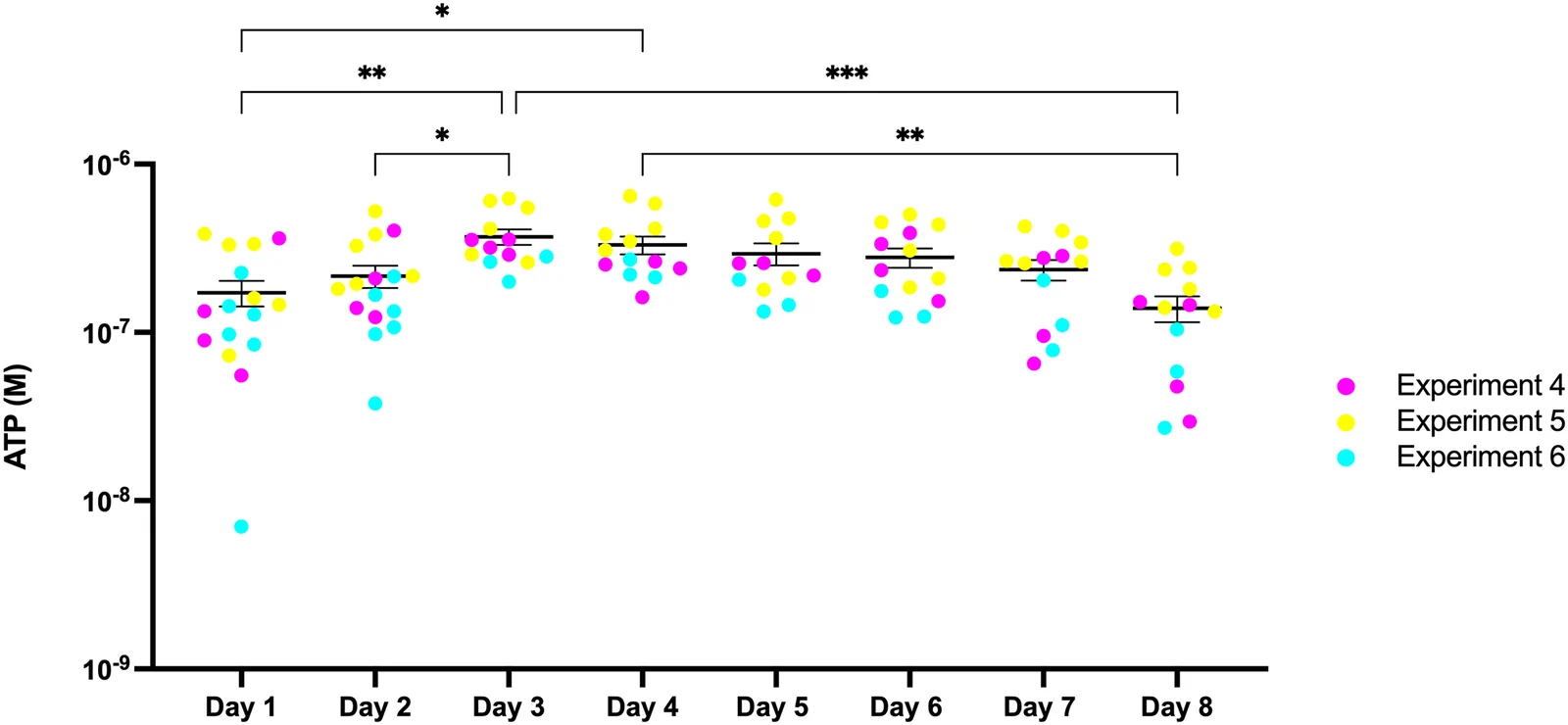
ATP quantification in individual sections following homogenization, over up to 8 days of culture across 3 independent experiments (n = 3 to 6 per experiment) Data are represented as mean ± SEM. ∗ p < 0.05; ∗∗ p < 0.01; ∗∗∗ p < 0.001.

**Figure 4:**
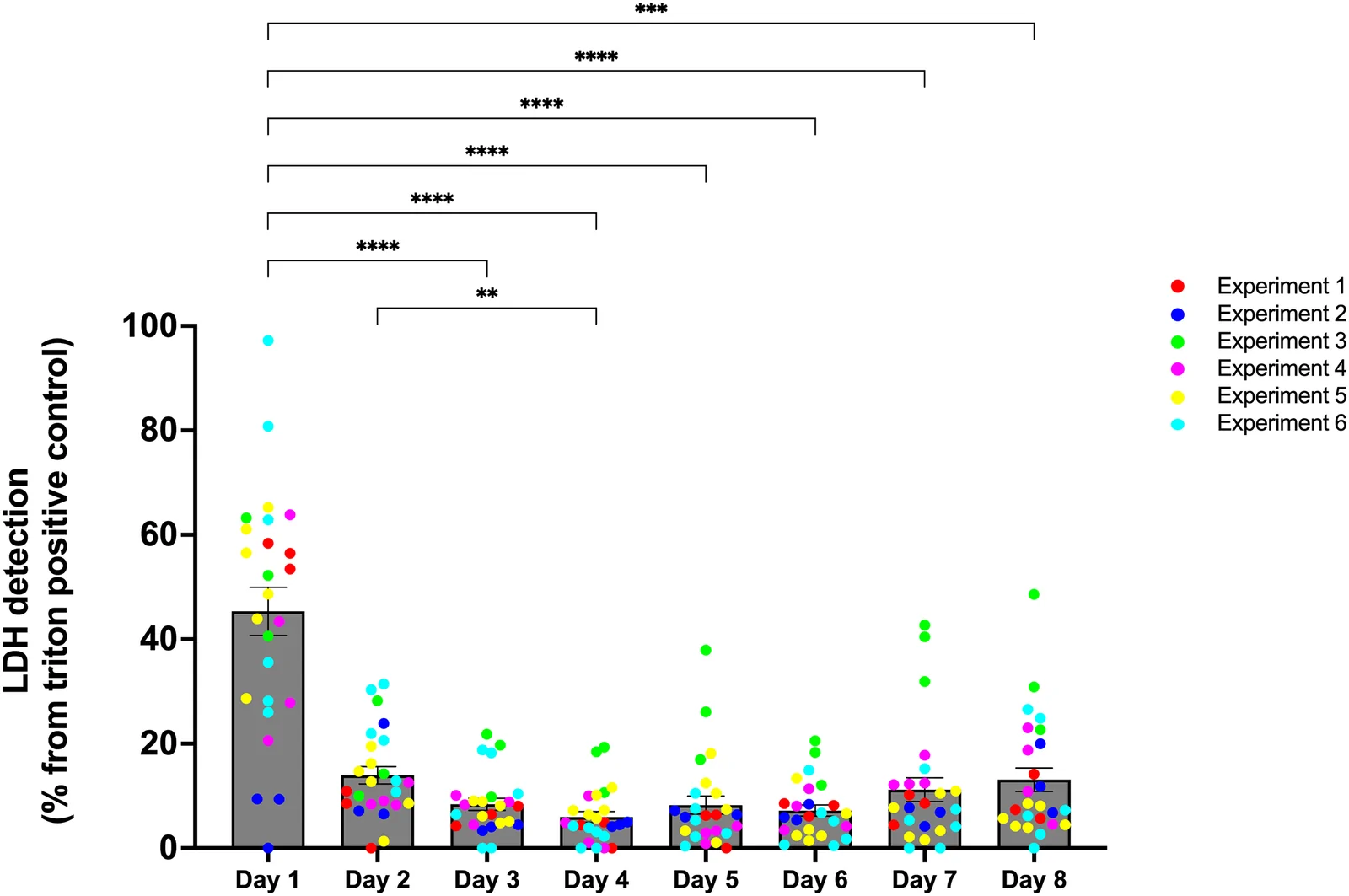
LDH detection in culture media over up to 8 days of culture across 6 independent experiments (n = 3 to 6 per experiment) Detection values are expressed as a percentage of the maximum detection recorded in positive controls (2% Triton X-100) on day 1 of culture. Data are represented as mean ± SEM. ∗∗ p < 0.01; ∗∗∗ p < 0.001; ∗∗∗∗ p < 0.0001.

ATP quantification revealed an initial reduction in cellular energy metabolism during the first two days of culture, attributable to the PCSS generation process itself. Intracellular ATP concentrations subsequently stabilized until day 7, before showing a marked decline at day 8 (Figure 3). LDH detection in the culture medium showed a significant increase during the first two days of culture, due to tissue stress induced by the generation process, and then decreased and stabilized from day 3 onward. A non-significant trend toward increased LDH release is observed at day 8 (Figure 4). These findings were consistent with and inversely correlated to ATP quantification. Indeed, a significant inverse correlation was observed between mean daily LDH detection and ATP quantification (r = −0.76, p = 0.03, non-parametric Spearman correlation). Together, these data suggested that PCSS viability can be monitored over time through LDH measurement in the culture medium alone.

The PCSS dimensions described here (10 × 20 × 0.5 mm) supported satisfactory viability and compatibility with all downstream procedures described in this study. This size yielded sufficient quantities of RNA from individual PCSS, and of protein when PCSS are pooled in pairs according to the extraction protocol described above (mean [*±SEM]* RNA concentration: 52.8 [*±4.0]* ng/μL in 40 μL; mean protein concentration: 1,727 [*±105.6]* μg/mL in 100 μL, see Table S1 for more details). Moreover, pooling two PCSS for cell dissociation generated an average yield of 226,000 cells (from 90,000 to 129,000 cells per PCSS) with preserved viability of 83–89% (Table 1). The combined RNA/protein extraction and dissociation protocols described here are therefore compatible with a range of downstream applications including qRT-PCR, western blotting and flow cytometry.

**Table 1:** Results of cellular count using Acridine Orange/Propidium Iodide staining after increasing number of PCSS dissociation (Day 1)

Number of PCSS	Cell count	Viability	Size (μm)
1	129000	83%	11,5
2	226000	88%	11,8
3	271000	89%	11,7
4	475000	87%	12,4

**Figure 5:**
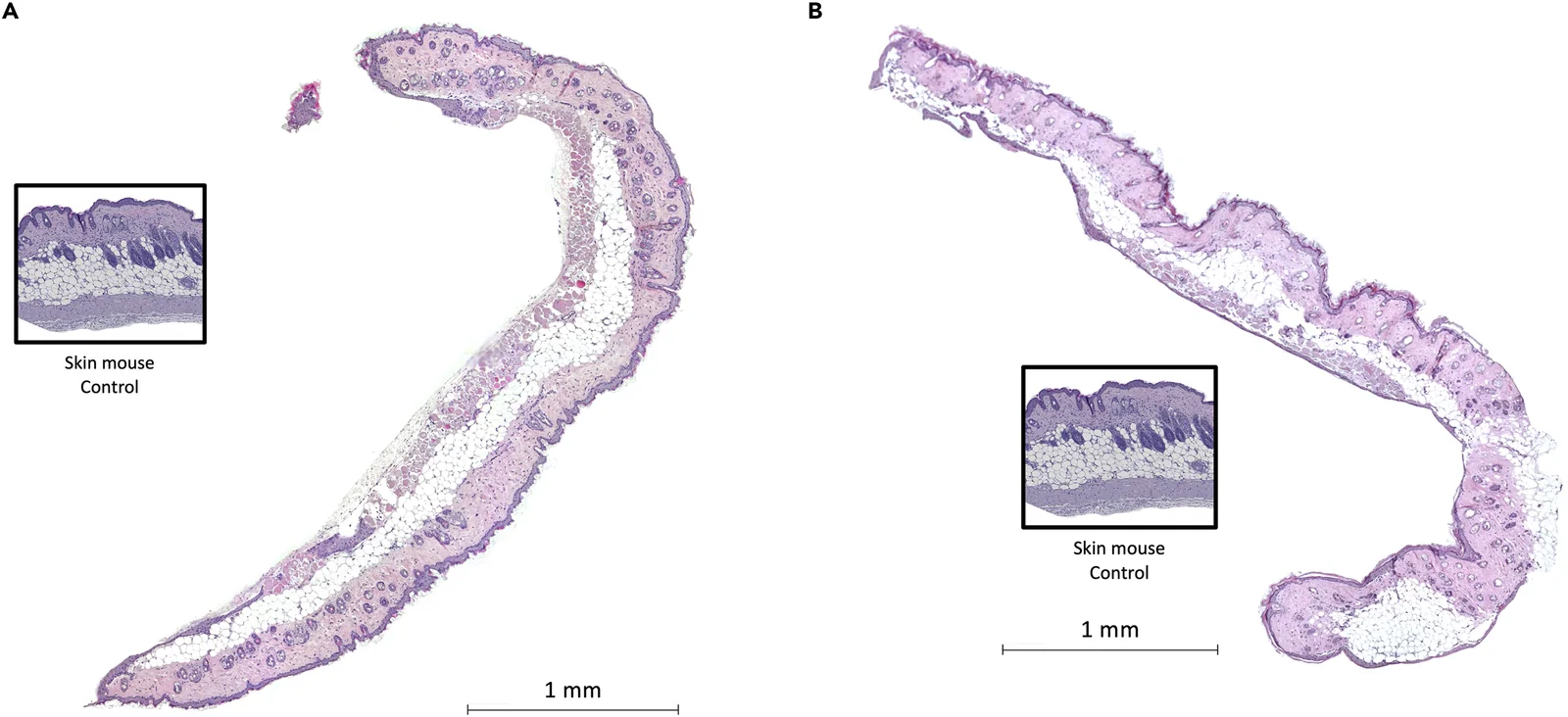
Hematoxylin and eosin staining of PCSS at various timepoints (×10 magnification) (A) Whole PCSS after 2 days of culture - Experiment 2; and (B) Whole PCSS after 6 days of culture - Experiment 3; with control BALB/c mouse skin for comparison.

**Figure 6:**
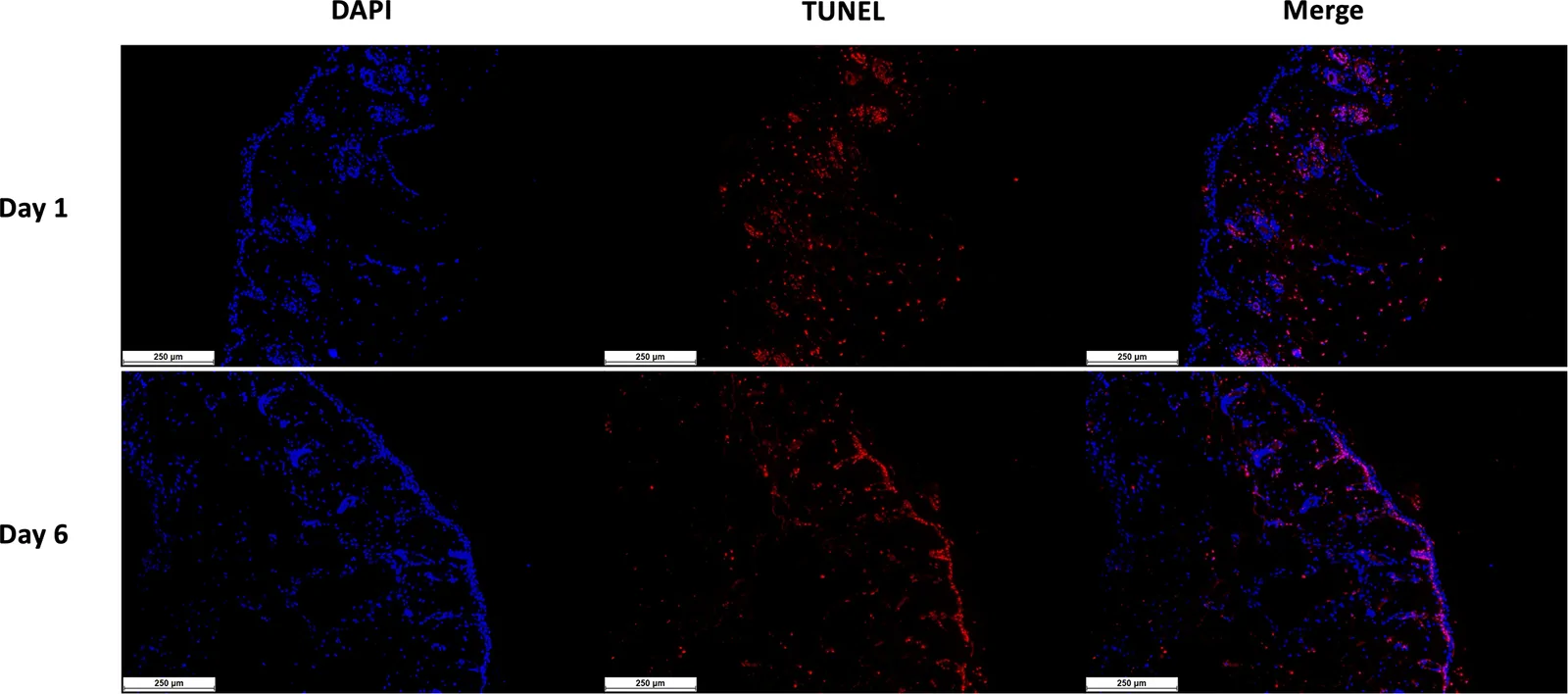
Immunofluorescence staining of nuclei with DAPI (blue) and apoptotic nuclei with TUNEL (red); PCSS at ×10 magnification after 1 day and 6 days of culture (Experiment 3)

Histological analyses confirmed the preservation of murine skin architecture. Progressive epithelial desquamation was observed from day 6 of culture, suggestive of selective keratinocyte death, alongside progressive disorganization of the overall skin architecture (Figure 5). TUNEL fluorescence analysis at day 1 of culture revealed localized cell death within circular dermal structures consistent with hair follicles due to both the PCSS generation process and prior depilation. From day 6 onward, cell death was observed at the periphery of the sections within the epidermal layer, consistent with the findings described above (Figure 6).

Taken together, these data confirmed the preserved viability of PCSS for up to 6 days of culture and demonstrated their compatibility with multiple analytical techniques.

## Quantification and statistical analysis

ATP Standard 4PL regression curve equation and ATP concentrations from luminescent signal were determined using the “[Agonist] vs. response – Variable slope (four parameters)” equation from the nonlinear regression models using GraphPad.

Results of LDH detection and ATP measurements are expressed as mean ± standard error of the mean (SEM). Day-by-day comparisons were performed using ordinary one-way ANOVA.

Correlation between LDH and ATP means was assessed using Spearman correlation test.

Data analyses and graphs were performed using the GraphPad Prism version 10.1.1 (GraphPad Software, La Jolla, CA).

Threshold for statistical significance was set to p < 0.05.

## Limitations

This protocol addresses the sectioning challenges typically encountered during PCSS generation, which result from the bio-mechanical properties of skin. The results presented have been validated using fresh skin samples from inbred BALB/c mice. Reproducibility across different genetic backgrounds or with non-fresh tissue has not been assessed. Furthermore, PCSS generation and characterization have only been performed on mouse skin. Human PCSS have been described in literature using a different protocol, involving smaller skin biopsies with a thinner dermal layer from patients with cutaneous fibrosis.^4^

A 20 × 10 mm skin sample yields approximately 40 PCSS. Variability between the first and last sections from the same sample may exist, although this was not observed during our experiments. If more than 40 PCSS are required, multiple specimen holders may be needed, each constituting an independent sectioning run that could introduce inter-holder variability.

The culture conditions described in this protocol do not support preservation of tissue architecture, cellular diversity, and energy metabolism beyond 6 days. Culture condition modifications may be required if longer culture duration is needed.

## Troubleshooting

### Problem 1

The Compresstome™ blade pushes rather than cuts through the skin sample (related to sub-steps 3, 5, and 10).

### Potential solution

• Sub-steps 2 to 5 are critical. These steps overcome the intrinsic resistance and elasticity of skin and are essential for successful sectioning. An excessively thick or elastic hypodermis may impair sectioning, it should be removed beforehand.^5^ Ensure the skin sample lies completely flat on the foam pad without creasing, and that both foam pads of the sandwich firmly hold the sample in place.
• Ensure that the adhesive is evenly distributed across both foam pads and the skin sample, and that liquid agarose is uniformly distributed throughout the metal insert.
• Sub-step 8 is also important: the skin sample must be oriented perpendicular to the Compresstome™ blade (see Figure 2G for optimal orientation).^6^ Published data confirm the importance of sectioning temperature in histology and PCSS generation. Ensure sectioning is performed in a temperature-controlled environment and that the PBS in the collection trough is maintained at 4°C.
• If PCSS generation becomes progressively more difficult, empty the collection trough and replace with fresh PBS at 4°C. On the manual Compresstome™ model used in this protocol, rotation of the micrometer screw causes progressive rotation of the specimen holder piston, regularly verify that the skin sample remains correctly oriented.

### Problem 2

PCSS becomes contaminated during culture (related to the PCSS generation steps).

### Potential solution

• Ensure that skin sample preparation is carried out under the strictest possible sterile conditions. House animals in a Specific Pathogen-Free facility and collect the skin sample as aseptically as possible following disinfection of the animal.
• Prepare the skin sample and perform Compresstome™ sectioning within a dedicated biosafety cabinet. Wear a face mask when handling the skin sample and avoid laboratories with air conditioning.
• PBS used for agarose melting and in the Compresstome™ collection trough may be supplemented with antibiotics.
• The culture medium may also be supplemented with additional antimicrobial agents, such as amphotericin B (0.5 μg/mL) or ciprofloxacin (4 μg/mL).

### Problem 3

PCSS adhere to pipette tips or well walls, or retract after several days of culture (related to sub-steps 12–13 and step “Samples collection and PCSS maintenance in culture”).

### Potential solution

• To optimize medium collection, tilt the culture plate and carefully aspirate the medium from the upper edge, allowing the PCSS to settle gently to the bottom of the well without obstructing medium retrieval. Alternatively, gently collect the medium from the bottom of the well, keeping the PCSS above the pipette tip end.
• To prevent PCSS adherence to pipette tips, pre-coat tips with FBS, or use low-retention pipette tips (e.g., Axygen®).
• Progressive retraction of PCSS was observed during experiments (see Figure S2). This retraction was variable, did not appear to be influenced by the time elapsed before sectioning, and did not affect ATP quantification or LDH detection results.

### Problem 4

Protein pellet resolubilization is incomplete or fails following TRIzol™ extraction (related to sub-step 30p).

### Potential solution

• Protein resolubilization difficulties are common after TRIzol™ extraction, particularly with tissues rich in keratins and collagens, such as skin. The protocol described here has been adapted from Ziros et al. for use with PCSS.^7^
• Repeat steps 30i, 30j, and 30k systematically, even if the pellet appears white, to ensure complete removal of residual phenol from the protein pellet.
• An alternative resolubilization solution described in the literature (such as 8 M urea/2 M thiourea) has demonstrated superior resolubilization efficiency and may be used as a substitute; however, this solution is incompatible with the Pierce™ BCA protein assay and is less suitable for western blot applications.^8^^,^^9^ In our experience, this resolubilization solution performs well for downstream proteomics applications by mass spectrometry.
• Incubate the sample at 50°C in a water bath or heat block for 3 to 5 min to improve resolubilization. Sonication may also be used to mechanically aid pellet solubilization. However, in our experience, both approaches may compromise protein quality and therefore require further optimization before routine use.

### Problem 5

Cell yield and/or viability following PCSS dissociation are abnormally low (related to sub-step 39).

### Potential solution

• Ensure that PCSS are cut into 1 to 2 mm pieces and fully submerged in the collagenase/dispase solution. Be careful not to let PCSS adhere to the tube walls (see troubleshooting 3), which can impair subsequent dissociation.
• Dissociation efficiency may be improved by incorporating a mechanical dissociation step (such as vortexing or a dedicated device e.g., MACS Tissue Dissociation Kits, Miltenyi Biotec®) to increase cell yield. Note that aggressive mechanical dissociation may adversely affect cell viability.
• Increasing the concentration of dissociation enzymes may improve cell yield; however, this may compromise the detection of surface markers in downstream flow cytometry applications, particularly those of resident cutaneous immune cells.

### Problem 6

Histological sections are incomplete or damaged (related to sub-steps 40 and 42).

### Potential solution

• Correct orientation of the PCSS during fixation in paraformaldehyde and subsequent paraffin embedding is essential for downstream histological analysis. In both sub-steps, ensure the PCSS is positioned as flat as possible on the foam pad or at the base of the mold.
• PCSS retraction during culture may complicate orientation at sub-step 40. Carefully unfold the PCSS without causing damage and orient it on the foam pad. Confirm linearity of the epidermis to verify the PCSS is not twisted (see Figure S3).
• While trimming the paraffin block, regularly check whether the PCSS is approaching the block surface. If so, continue trimming gently using 4 to 10 μm sections to avoid excessive tissue loss that would occur with 20 μm trimming sections.
• Once the paraffin section has been placed on the slide and transferred to the hot plate, drain the water as soon as the paraffin has flattened. Prolonged exposure to the hot plate risks dermis-epidermis separation, a risk that increases with longer culture durations due to progressive tissue fragility.

## Resource availability

### Lead contact

Further information and requests for resources and reagents should be directed to and will be fulfilled by the lead contact, David Launay (david.launay@univ-lille.fr).

### Technical contact

Questions about the technical specifics of performing the protocol should be directed to and will be fulfilled by the technical contact, Manel Jendoubi (manel.jendoubi@univ-lille.fr).

### Materials availability

This study did not generate new unique reagents.

### Data and code availability

This study did not generate or analyze datasets and codes.

## Acknowledgments

We would like to thank Dr. Muriel Pichavant for her invaluable assistance at the inception of this project and for her continued support and availability throughout its development.

We would also like to thank Léo Cavarec for critical reading of the protocol and photographic support. This work received no specific grant from any funding agency in the public, commercial, or not-for-profit sectors.

## Author contributions

**N.P.,** conceptualization, methodology, investigation, formal analysis, writing – original draft, and writing – review and editing; **M.J.,** conceptualization, resources, investigation, methodology, writing – original draft, and writing – review and editing; **B.D.**, methodology and writing – original draft; **F.M.**, methodology and writing – original draft; **A.L.**, methodology and writing – original draft; **M.D.**, methodology and writing – original draft; **L.D.,** resources, project administration, and writing – original draft; **S.D.**, conceptualization, resources, supervision, validation, project administration, and writing – review and editing; **D.L.**, conceptualization, resources, supervision, validation, project administration, and writing – review and editing; **S.S.**, conceptualization, methodology, resources, supervision, project administration, writing – original draft, and writing – review and editing.

## Declaration of interests

The authors declare no competing interests.
