# Supplementary material for: Protocol for the generation, viability assessment, and downstream processing of murine precision-cut skin slices
Source: STAR Protoc. 2026 Sep 10;7(3):104828. doi: 10.1016/j.xpro.2026.104828 (PMC13584097; doi:10.1016/j.xpro.2026.104828)
Supplement: Supplementary file 1 [file mmc1.pdf]

Supplementary data

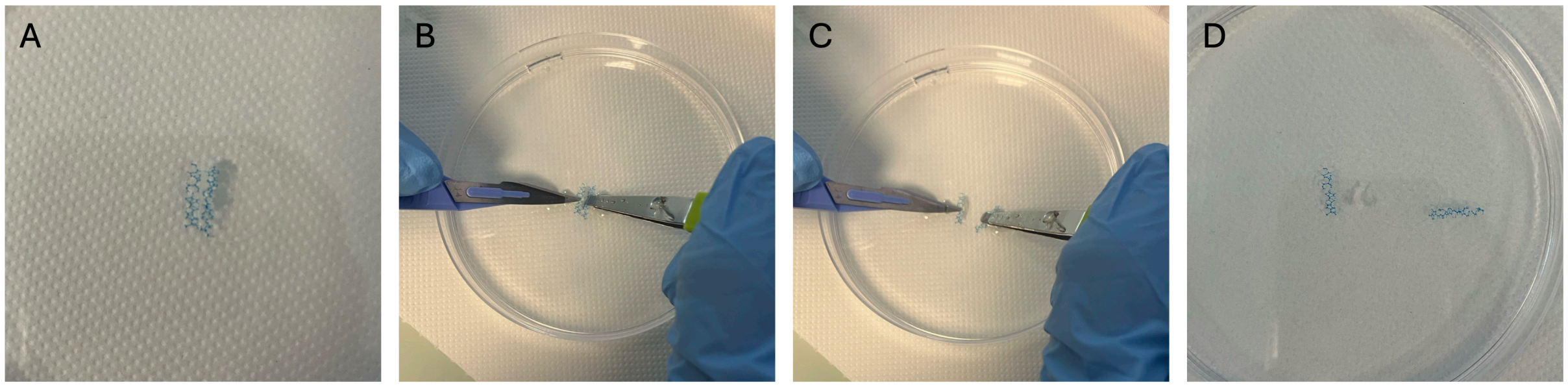

Figure S1: Illustration of different steps of removing PCSS from agarose embedding and foam pad sandwich, related to substep 10c:

- *(A) Skin sample within foam pad sandwich and agarose in 37°C PBS.*
- *(B) Remove genteelly the first foam pad to free the PCSS from the sandwich.*
- *(C) Catch the PCSS with the forceps and pull softly and slowly.*
- *(D) PCSS is free in PBS and can be put in the culture dish.*

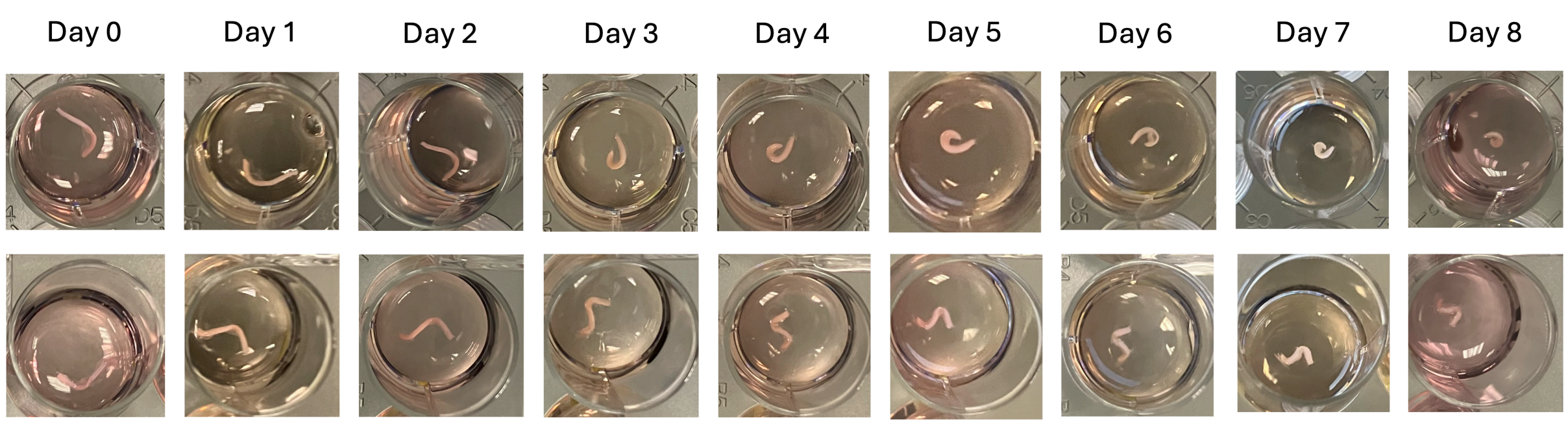

Figure S2: Progressive retractation of PCSS along culture (Experimentation 3), related to Troubleshooting 3.

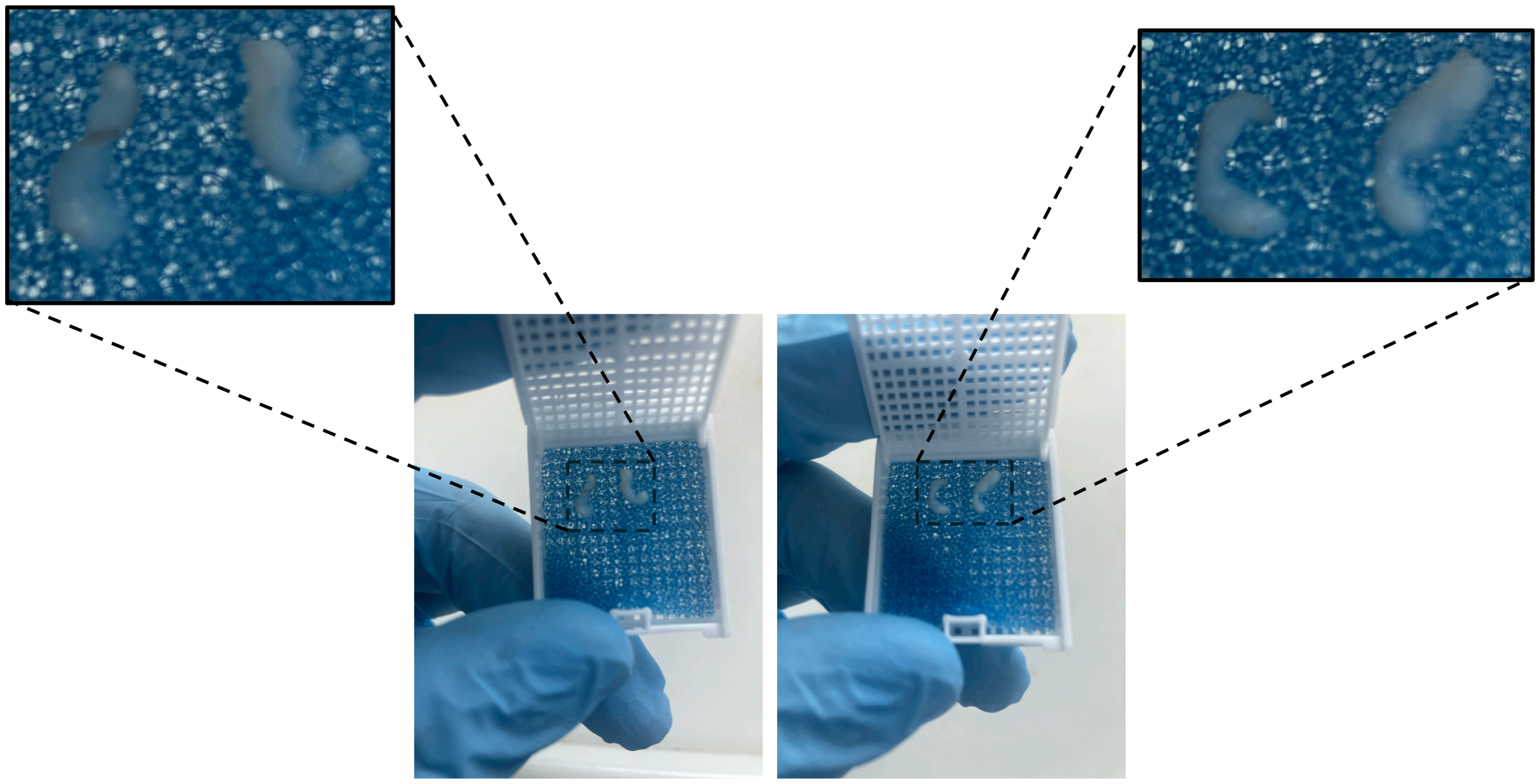

Figure S3: Twisting PCSS with nonlinear epidermis positioned on the foam pad, related to Troubleshooting 6.

Table S1: Results of RNA quantification with NanoDrop™ DS-11 and protein quantification using Pierce™ BCA Protein Assay Kit after TRIzol™ combined extraction.

Day	Experimentation	PCSS n°	RNA (ng/µL)	260/280 nm	260/230 nm	Protein (µg/mL)
Day 0	Exp 2	1	19.153	1.855	1.699	1423
		2	19.489	1.822	0.643	
	Exp 3	1	17.679	1.866	1.559	1678
		2	14.894	1.902	1.721	
Day 1	Exp 2	1	30.179	1.907	1.875	2468
		2	35.746	1.984	2.001	
	Exp 3	1	53.260	2.002	2.101	1358
		2	50.612	1.993	2.098	
Day 2	Exp 2	1	68.155	2.005	2.018	1873
		2	95.860	2.009	2.010	
	Exp 3	1	91.250	2.022	2.216	2043
		2	80.703	2.044	2.142	
Day 3	Exp 2	1	51.298	2.000	1.708	1648
		2	37.936	1.884	1.561	
	Exp 3	1	81.225	1.995	2.07	1728
		2	84.338	2.057	2.116	
Day 4	Exp 2	1	65.453	2.039	2.182	1538
		2	34.342	1.858	1.692	
	Exp 3	1	69.386	2.025	2.124	2338
		2	84.676	2.062	2.039	
Day 5	Exp 2	1	22.974	1.903	1.917	1603
		2	42.258	1.979	1.862	
	Exp 3	1	55.358	2.004	2.073	2563
		2	36.031	1.952	2.012	
Day 6	Exp 2	1	58.163	2.002	2.081	2228
		2	30.805	1.935	2.008	
	Exp 3	1	46.429	1.888	1.590	1648
		2	51.576	2.010	1.894	
Day 7	Exp 2	1	55.643	1.994	2.023	1123
		2	49.681	1.985	2.065	
	Exp 3	1	61.010	1.907	1.874	1533
		2	56.074	1.995	1.795	
Day 8	Exp 2	1	116.09	1.422	1.824	988
		2	50.086	1.947	1.934	
	Exp 3	1	47.285	1.743	1.215	1308
		2	35.650	1.580	1.420	
